# Influenza Infects Lung Microvascular Endothelium Leading to Microvascular Leak: Role of Apoptosis and Claudin-5

**DOI:** 10.1371/journal.pone.0047323

**Published:** 2012-10-24

**Authors:** Susan M. Armstrong, Changsen Wang, Jayesh Tigdi, Xiaoe Si, Carlo Dumpit, Steffany Charles, Asela Gamage, Theo J. Moraes, Warren L. Lee

**Affiliations:** 1 Institute of Medical Science, University of Toronto, Toronto, Canada; 2 Keenan Research Centre of the Li Ka Shing Knowledge Institute of St. Michael’s Hospital, Toronto, Canada; 3 Hospital for Sick Children, Toronto, Canada; 4 Division of Respirology and Interdepartmental Division of Critical Care Medicine, Faculty of Medicine, University of Toronto, Toronto, Canada; Lovelace Respiratory Research Institute, United States of America

## Abstract

Severe influenza infections are complicated by acute lung injury, a syndrome of pulmonary microvascular leak. The pathogenesis of this complication is unclear. We hypothesized that human influenza could directly infect the lung microvascular endothelium, leading to loss of endothelial barrier function. We infected human lung microvascular endothelium with both clinical and laboratory strains of human influenza. Permeability of endothelial monolayers was assessed by spectrofluorimetry and by measurement of the transendothelial electrical resistance. We determined the molecular mechanisms of flu-induced endothelial permeability and developed a mouse model of severe influenza. We found that both clinical and laboratory strains of human influenza can infect and replicate in human pulmonary microvascular endothelium, leading to a marked increase in permeability. This was caused by apoptosis of the lung endothelium, since inhibition of caspases greatly attenuated influenza-induced endothelial leak. Remarkably, replication-deficient virus also caused a significant degree of endothelial permeability, despite displaying no cytotoxic effects to the endothelium. Instead, replication-deficient virus induced degradation of the tight junction protein claudin-5; the adherens junction protein VE-cadherin and the actin cytoskeleton were unaffected. Over-expression of claudin-5 was sufficient to prevent replication-deficient virus-induced permeability. The barrier-protective agent formoterol was able to markedly attenuate flu-induced leak in association with dose-dependent induction of claudin-5. Finally, mice infected with human influenza developed pulmonary edema that was abrogated by parenteral treatment with formoterol. Thus, we describe two distinct mechanisms by which human influenza can induce pulmonary microvascular leak. Our findings have implications for the pathogenesis and treatment of acute lung injury from severe influenza.

## Introduction

Influenza remains one of the most common infectious causes of death in the Western world [1]. Its high genetic variability and development of resistance to antiviral medications have made the development of novel therapies a priority. Life-threatening infections with influenza are characterized by acute lung injury (ALI), a syndrome of increased pulmonary microvascular permeability that leads to hypoxia and respiratory failure [2]. The pathogenesis of the pulmonary microvascular leak in these cases is not known. We hypothesized that ALI that occurs in severe influenza could be a result of infection of the lung microvascular endothelium.

There is little literature on this notion [3], [4], yet several observations suggest that it is plausible. Although respiratory epithelial cells are the main targets of human influenza, human endothelial cells are known to express α(2,6)-linked sialic acid residues, the receptor for the virus [5], [6]. The expression of these sialic acid linkages increases when endothelial cells are stimulated with cytokines, as might occur in serious infections [7]. Second, other pathogenic viruses (e.g. avian influenza) can infect endothelial cells [8], [9]. Third, recent data suggest that the lung endothelium may play a critical role in regulating cytokine production after influenza infection [10]. Finally, an infection of the respiratory endothelium is plausible given the close proximity of the respiratory epithelium. However, whether and how infection of human lung microvascular endothelium by influenza can itself cause barrier dysfunction remains to be elucidated.

Endothelial barrier function depends on the integrity of intercellular junctions which bridge adjacent cells; of these, adherens junctions and tight junctions are the most important [11], [12]. Tight junction strands form a physical barrier preventing the passage of solutes between cells and are composed of numerous proteins, claudins and occludins being the major components [13], [11]. While over 20 claudins have been described, their tissue distribution varies greatly [14]; only claudin-5 is expressed predominantly in endothelial cells of all organs and is especially enriched in the lung [11]. Interestingly, its deletion leads to a size-selective defect in the blood-brain barrier of knockout mice [15]. Similarly, degradation of claudin-5 was associated with an increase in dermal microvascular permeability in an in vitro model of malaria [16] and knockdown of claudin-5 in human umbilical vein endothelium caused a decrease in endothelial monolayer electrical resistance [17]. In a mouse model of ALI, a compensatory increase in claudin-5 levels was observed in mice that were resistant to vascular leak [18]. Taken together, these results suggest that claudin-5 is important in regulating endothelial permeability. In contrast to tight junctions, in adherens junctions the major constituent is VE-cadherin [12]. Inflammatory mediators can induce the internalization and/or degradation of plasmalemmal VE-cadherin, which is sufficient to increase endothelial permeability [19].

In addition to modification of intercellular junctions, leak can occur from remodeling of the actin cytoskeleton characterized by a loss of cortical actin and an increase in actinomyosin stress fibers [20]. Contraction of these stress fibers leads to a change in cell shape and the formation of intercellular gaps. Finally, certain pathogens and inflammatory mediators may also induce pulmonary vascular leak by causing endothelial apoptosis or damage [21].

Determining whether lung endothelial barrier integrity is a target for human influenza is an important issue with implications for both the pathogenesis and treatment of this infection. For instance, enhancement of endothelial barrier integrity might represent an attractive therapeutic option since it does not target the virus and would theoretically be less susceptible to viral mutation. In this study, we determined whether human influenza is capable of infecting the pulmonary microvascular endothelium and whether infection leads to the loss of barrier integrity. Finally, we determined the mechanisms of the increase in vascular leak and developed an animal model of ALI from influenza.

## Materials and Methods

### Ethics Statement

All mouse experiments were performed in accordance with the regulations of the Canadian Council on Animal Care and were approved by the Animal Care Committee of the Hospital for Sick Children (protocol #8911). Care was taken to minimize animal discomfort as per institutional guidelines. Thus, mice were anesthetized with isoflurane for intranasal instillation and were monitored up to two times a day post infection.

### Reagents

To measure permeability, fluorescein isothiocyanate (FITC)-dextran (MW 70 kDa, Invitrogen) was added at a concentration of 50 µg/mL for 40 minutes. To inhibit apoptosis, cells were treated with 80 µM ZVAD-FMK (Enzo Life Sciences) for 24 hours. For proteasome inhibition, cells were treated with 20 µM MG-132 (Calbiochem) for 6 hours. The cAMP analogue, pCPT-cAMP (Sigma) was added at a concentration of 0.25 mg/mL for 24 hours prior to influenza infection and again at the start of the infection. Marimastat (Santa Cruz) was added at a concentration of 100 µM for 24 hours to inhibit matrix metalloproteases. To induce claudin-5 expression in vitro, formoterol (Sigma) was added at the indicated concentrations for 24 hours. To evaluate the effect of influenza binding, recombinant hemagluttinin (HA; Immune Technology Corp.; catalogue no. IT-003-00418ΔTMp) from H3N2 was used at the indicated doses.

### Cells and Influenza Infection

Primary human lung microvascular endothelial cells (HMVECs) obtained from Lonza were cultured in EBM-2 media with the recommended supplements and used in passages 6–9. Primary C57BL/6 mouse lung microvascular endothelial cells were obtained from Cell Biologics (Chicago, IL) and were cultured with Mouse Endothelial Cell Medium with the recommended supplements. We used influenza A X31 (H3N2, originally a gift from Dr. Tania Watts) [22] since the H3N2 subtype is most commonly associated with complications and death [23], [24]; we also used a clinical isolate (H3N2, from Dr. Susan Richardson) to confirm our key findings. The virus was added to cells in serum-free media. After one hour, 0.5% serum was added. All infections were for 24 hours unless otherwise indicated. To create replication-deficient virus, influenza was exposed to UV light for 10 minutes and lack of replication was confirmed as indicated below. Cells were infected at the apical surface unless otherwise noted. The amount of virus was quantified both by plaque forming units and by hemagglutinin units (HAU) using published protocols [25].

### 50% Tissue Culture Infective Dose (TCID50)

After treatment of endothelial cells with influenza (25 HAU/100 000 cells) for 1 hour, the supernatant was aspirated and cells were incubated in fresh serum-free media. At each timepoint, the cells were trypsinized, then harvested and centrifuged. The supernatant, after centrifugation, was then diluted 10-fold and put in a 96-well plate containing MDCK cells. Viral titer was then determined by the Reed and Muench method by quantifying red blood cell agglutination by influenza, as previously described [26].

### Real-time PCR

Cells were treated with influenza for 1 hour. The supernatant was aspirated and the cells were washed twice with PBS and then incubated in serum-free media. The supernatant was collected for different timepoints and RNA was isolated using the QIAamp Viral RNA mini kit (Qiagen, Valencia, CA, USA); cDNA synthesis was carried out using the High-Capacity cDNA Reverse Transcription Kit according to the manufacturer’s instructions. For each sample, RNA was reverse-transcribed using T-Gradient Thermoblock (Biometra) according to the manufacturer’s directions. Q-PCR was conducted using Power SYBR Green PCR Master Mix (Applied Biosystems). cDNA was denatured at 95°C for 10 minutes followed by 40 cycles of 95°C for 15 seconds then 60°C for 1 minute. Q-PCR was performed with the ABI Prism 7900HT (Applied Biosystems), and the data were analyzed with SDS software v2.1 (Applied Biosystems) and Microsoft Excel 2003 (Microsoft). Relative gene expression was compared using the comparative C_T_ method [27]. Primer sets used for this study are as follows: Influenza A M1: (RT) 5′-TCTAACCGAGGTCGAAACGTA-3′; (Q-PCR) Forward 5′AAGACCAATCCTGTCACCTCTGA-3′; Reverse 5′-CAAAGCGTCTACGCTGCAGTCC-3′; 18s rRNA: (Q-PCR) Forward 5′-GATGGAAAATACAGCCAGGTCCTA-3′ and Reverse 5′-TTCTTCAGTCGCTCCAGGTCTT-3′. A fixed amount of cellular cDNA was added to each reaction so that expression of 18s RNA could be used as a reference.

**Figure 1:**
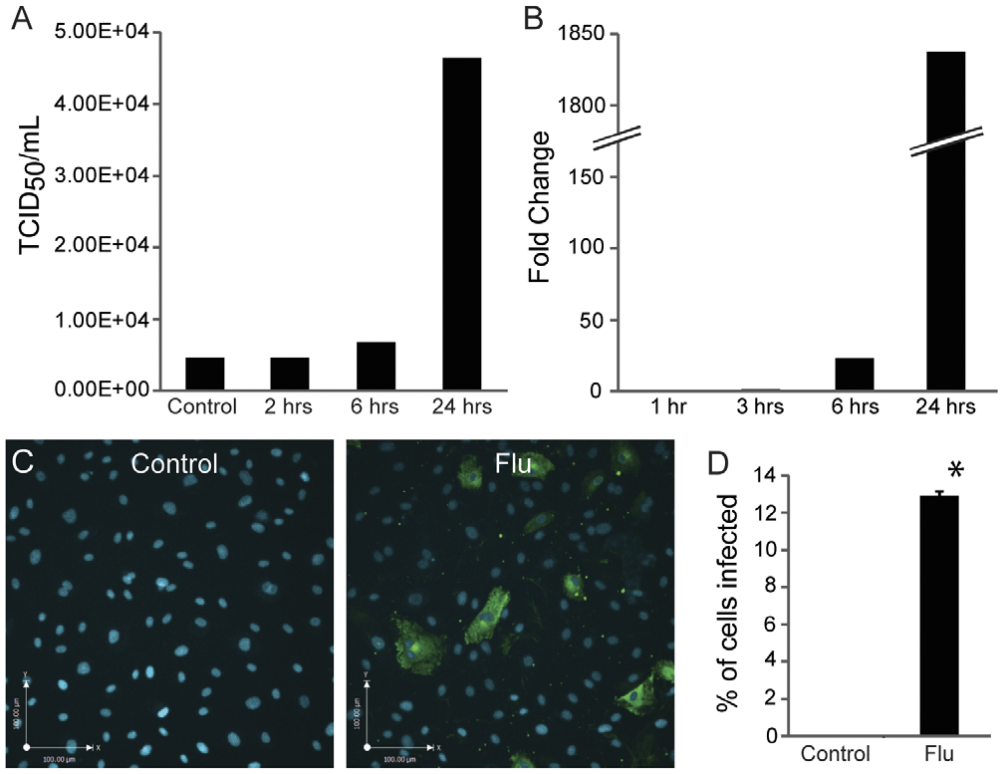
Human influenza replicates in primary lung microvascular endothelial cells. (A) Viral titer increases over time as shown by TCID_50_ assay. This assay quantitates the ability of influenza to agglutinate red blood cells after viral replication (see Materials and Methods section for further details). The initial influenza dose was 25 HAU/100 000 cells. The control group had no cells, received the same influenza dose, and was analyzed at 24 hours. Results are representative of 3 experiments. (B) qPCR showing the fold change in viral RNA for the influenza A M1 protein (see Materials and Methods for primers) over time. Results are representative of 3 experiments. (C–D) Immunofluorescent images (C) and quantitation (D) showing the percentage of cells infected by influenza after 24 hours. Influenza was given at 40 HAU/100 000 cells. Nuclei are stained with DAPI and viral nucleoprotein is shown in green. Images are representative of 3 experiments; data are mean and standard error, *p<0.05 for flu vs. control (uninfected cells).

### Immunofluorescence

For actin, VE-cadherin, p65, M1, and viral nucleoprotein (NP) immunostains, cells were fixed in 4% paraformaldehyde (PFA) for 1 hour at room temperature, incubated in 0.15% glycine for 10 minutes, and permeabilized in 0.1% Triton X-100 for 20 minutes. For the claudin-5, poly-ubiquitin (FK1), and LAMP1 immunostains, cells were fixed in methanol. After blocking, cells were treated with Alexa Fluor 488 Phalloidin (Molecular Probes) to stain actin. VE-cadherin, NP, and LAMP1 primary antibodies were from Santa Cruz Biotechnology, Claudin-5 was from Abcam, and Anti-influenza H3N2 M1 was from Thermo Scientific. Images were acquired by spinning disc confocal microscopy (Zeiss Axiovert 200 M microscope). Microscope settings were kept constant between conditions. All images were randomly chosen and were acquired as z-stack projections (z-interval 0.5 µm).

### Permeability Assay

HMVECs seeded on 0.4 µm-pore polyester transwells (Costar) coated with Attachment Factor (Invitrogen) were grown to confluency for 3–4 days. Baseline permeability to FITC-dextran was then measured as previously described [28]. As a complementary approach, the transendothelial electrical resistance (TEER) of endothelial monolayers was measured using the Endohm-12 (WPI, Florida). Cells were then treated with influenza at different multiplicities of infection (MOI; defined as the ratio of plaque forming units to endothelial cells) for 24 hours. Permeability to dextran and/or the TEER were then measured and compared to (pre-infection) baseline.

**Figure 2:**
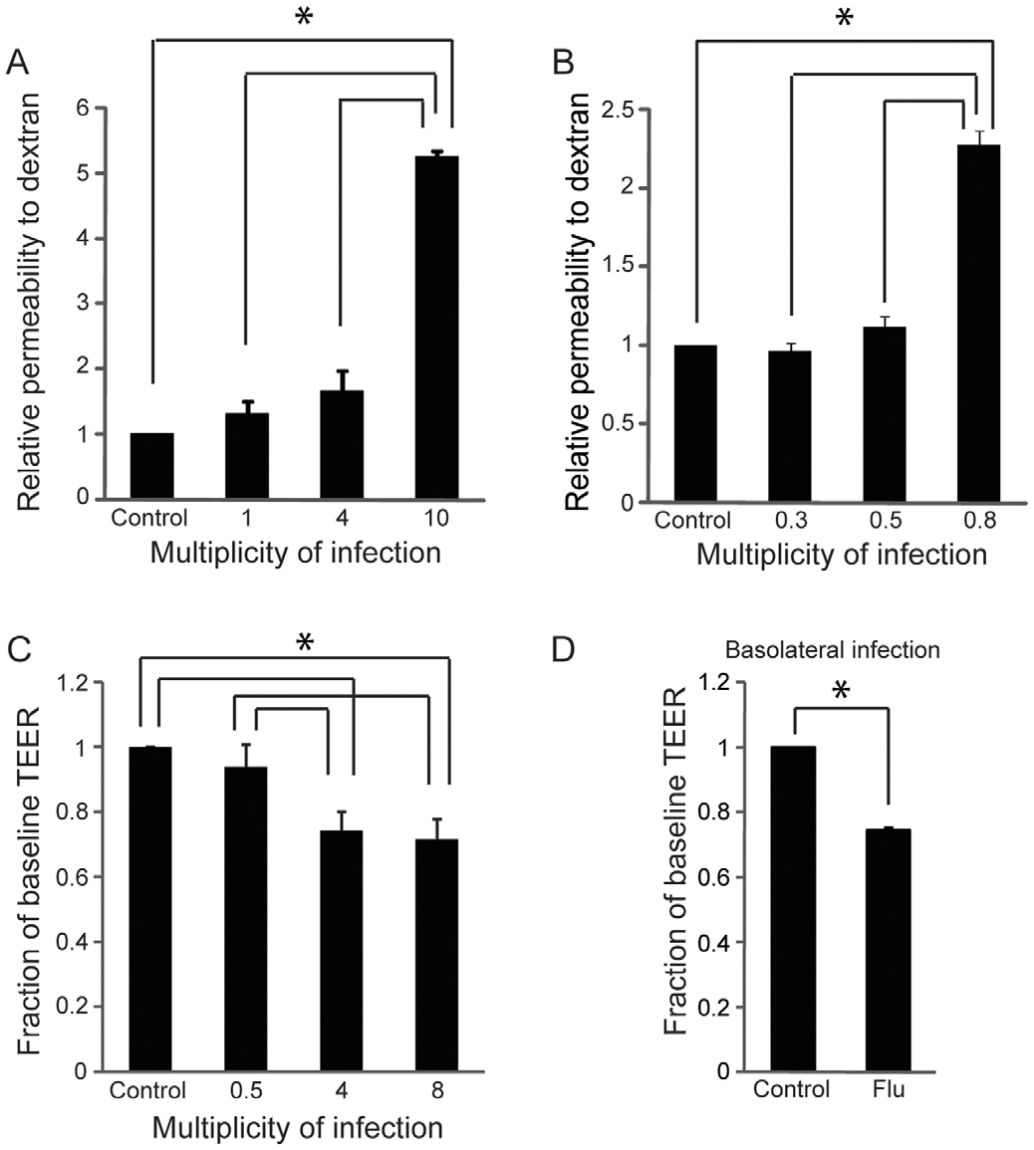
Human influenza induces lung endothelial permeability. (A) Influenza induces an increase in permeability to dextran in a dose-dependent fashion. Cells were infected at the indicated multiplicity of infection (MOI) for 24 hours before permeability to dextran was analyzed. Results (mean, SD) are experiments and are normalized to control, *p<0.05 versus control. (B) A clinical H3N2 influenza isolate was used to infect lung microvascular endothelium for 24 hours. Permeability to dextran was measured as in A. Results (mean, SD) are from 3 experiments and are normalized to control, *p<0.05. (C) Similar to A, except the transendothelial electrical resistance (TEER) was measured before and after infection. Results (mean, SE) are from 3 experiments and are normalized to control, *p<0.05. (D) Influenza induces endothelial permeability even after infection from the basal aspect. Human lung microvascular endothelium seeded on transwells was infected from the basal aspect of the transwell (MOI 8) and the change in TEER was measured 24 hours later. Results (mean, SD) are from 2 experiments and are normalized to control, *p<0.05.

**Figure 3:**
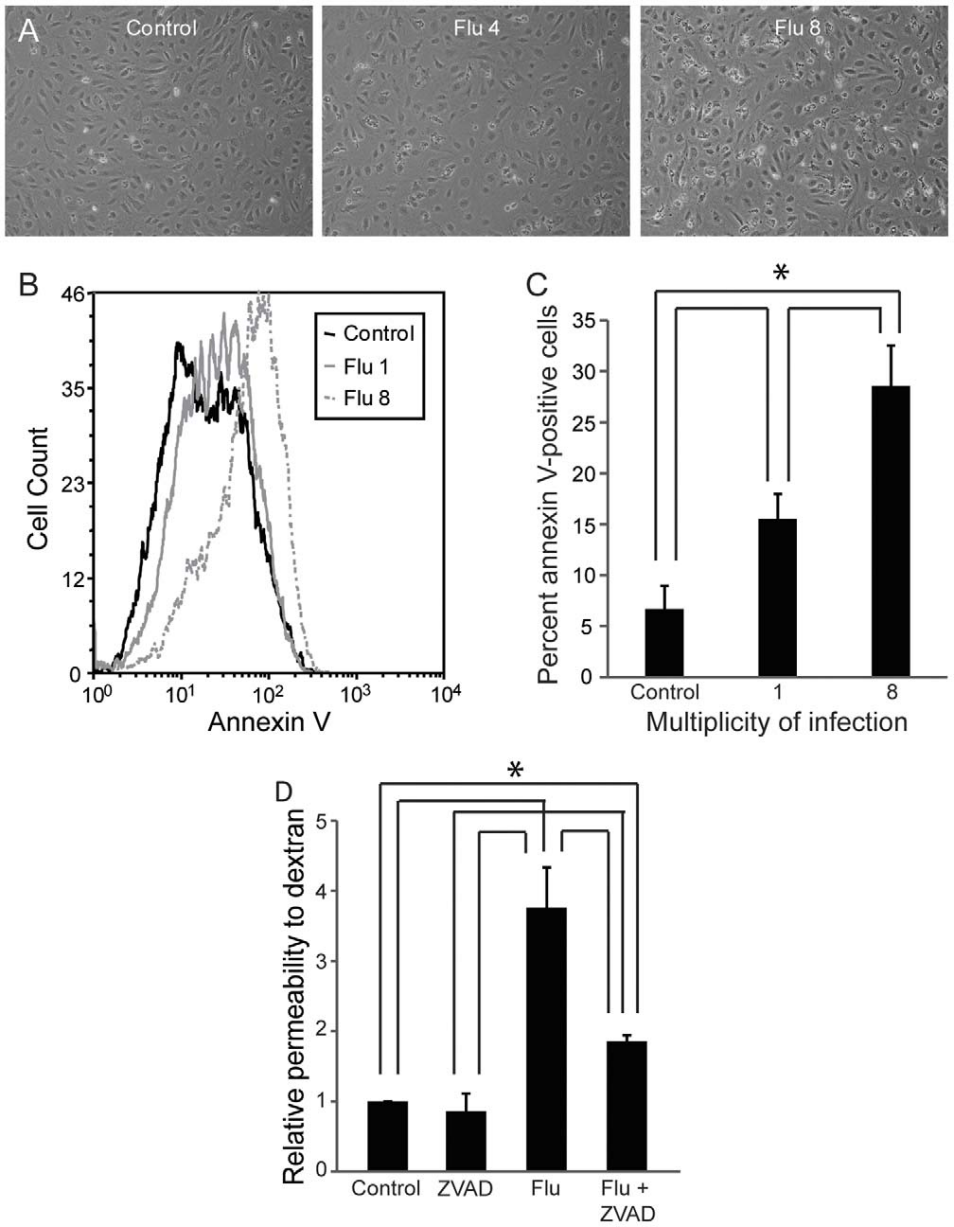
Human influenza induces lung endothelial apoptosis. (A) Influenza induces loss of endothelial cell viability, detected by phase contrast microscopy. Cells were infected for 24 hours at the indicated multiplicity of infection (MOI). Images were captured using a Nikon Eclipse and are representative of 3 experiments. (B) Influenza induces lung endothelial apoptosis as shown by flow cytometry. Cells were infected with influenza at the indicated MOI for 4 hours and binding of annexin V was measured. (C) The number of annexin V-positive cells increased significantly in a dose-dependent fashion. Y-axis is the percentage of cells that are positive for annexin V. Results (mean, SE) are from 4 experiments, *p<0.05. (D) ZVAD-FMK partially prevents the induction of influenza-mediated lung endothelial permeability. Endothelial cells on transwells were infected with influenza (80 HAU/100 000 cells) for 24 hours with or without 80 µM ZVAD-FMK. Permeability to dextran was measured. Results (mean, SE) are from 4 experiments and are normalized to control, *p<0.05.

### Detection of Apoptosis

Cells were prepared using the Annexin V-FITC Apoptosis Detection Kit (BioVision) according to the manufacturer’s instructions and analyzed by flow cytometry using a BD FACS Calibur cytometer (Becton Dickinson); cells were probed with Annexin V and propidium iodide to detect apoptosis and necrosis, respectively [29]. Data was analyzed using De Novo Software- FCS Express v 3.0.

### Western Blot

Lysates were prepared with lysis buffer (62.5 mM Tris-HCl pH 6.8, 2% SDS, 10% glycerol, 10 mM DTT) and separated using 10% polyacrylamide gels. Proteins were transferred to nitrocellulose membranes, blocked for 1 hour in 5% milk in TBS, and probed overnight with primary antibody at 4°C. After washing, blots were incubated with HRP-conjugated secondary antibodies for 1 hour, washed, and then visualized by advanced chemiluminescence (Amersham). Band intensity was quantified using Image J (NIH) and normalized to the loading control after background correction.

### Immunoprecipitation

Cell lysate (350 µg protein) in 1 mL of lysis buffer (1% SDS, 1 mM EDTA, and complete protease inhibitors (Roche)) was incubated with 25 µL of protein G beads (Thermo Scientific) at 4°C for 1 hour for pre-clearing. Antibody (1 µg of rabbit anti-claudin-5, Santa Cruz) was added to pre-cleared cell lysate with 25 µL of protein G beads for 1 hour at 4°C with rotation. The beads were then washed 3 times with lysis buffer (50 mM Tris-HCl, pH 8, 150 mM NaCl, 10 mM MgCl_2_, 1 mM EDTA, and 1% Triton X-100) and centrifuged at 5000 g at 4°C for 1 minute. The beads were boiled in SDS-PAGE loading buffer for 5 minutes. The supernatant was analyzed by western blot.

**Figure 4:**
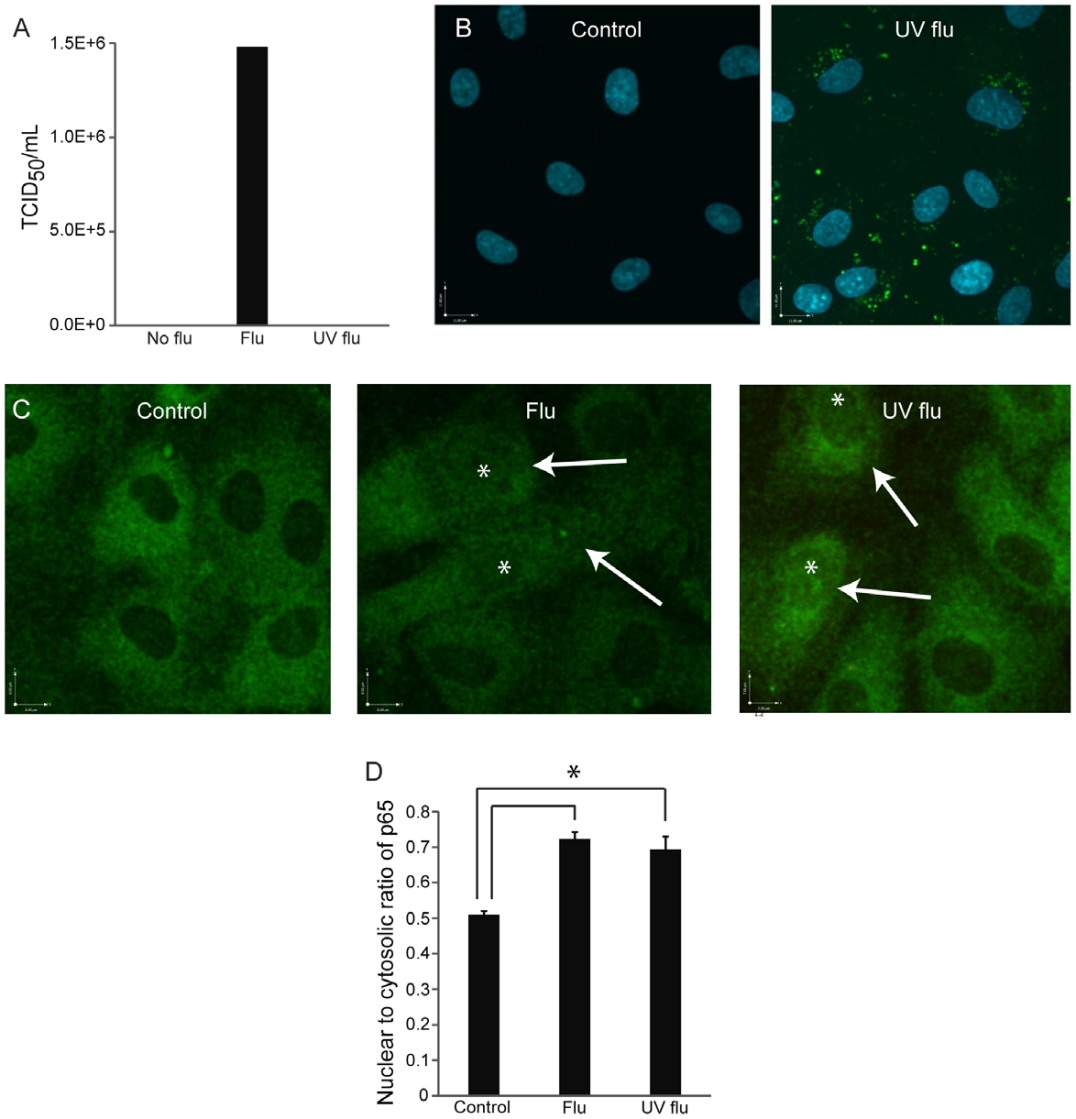
Characterization of replication-deficient virus. (A) Influenza irradiated with ultraviolet light (UV flu) cannot replicate. Influenza (40 HAU/100 000 cells) was irradiated with UV light for 10 minutes before viral titer was measured using the TCID_50_ assay as in Figure 1A. Results are representative of 2 experiments. (B) Viral proteins are expressed in endothelial cells infected with UV-irradiated influenza. Influenza was given at an MOI of 8 for 12 hours. Nuclei are stained with DAPI and viral nucleoprotein is shown in green. Images are representative of 2 experiments. Immunofluorescent images (C) and quantitation (D) showing the ratio of nuclear to cytosolic p65, a measure of NFκB activation, in cells infected by influenza after 12 hours. Influenza was given at an MOI of 8. Arrows indicate infected cells, while asterisks denote their nuclei. P65 is shown in green. Infected cells were identified by immunofluorescence for influenza viral protein M1 (not shown). Images are representative of 4 experiments; data are mean and standard error, *p<0.05 for live flu and UV flu vs. control (uninfected cells).

**Figure 5:**
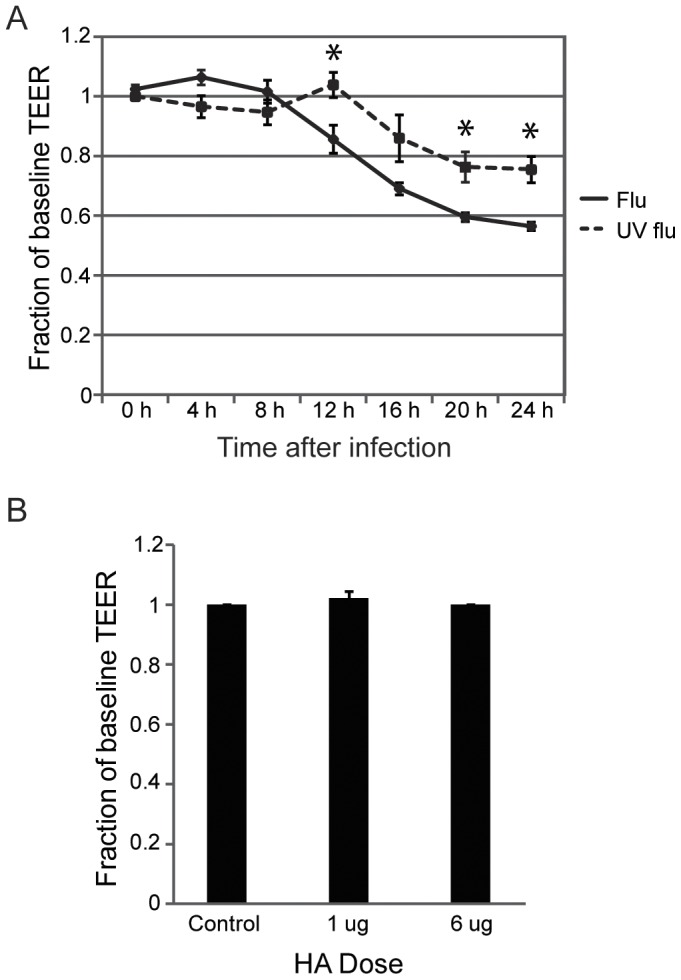
Replication-deficient virus induces lung endothelial permeability. (A) Replication-deficient influenza induces endothelial permeability to a lesser degree than live virus over time. Lung endothelium was infected with either live or replication-deficient (UV flu) influenza and the change in TEER was measured every 4 hours for 24 hours. Data for live flu (mean, SE) are from 4 experiments and for UV flu are from 5 experiments. All data are normalized to control, *p<0.05 for live vs UV flu. (B) Binding of influenza to endothelial cells is insufficient to produce leak. Lung endothelial cells were treated with indicated doses of hemagglutinin (HA) (Immune Technology Corp.) and the change in TEER was measured after 24 hours. Data (mean, SE) are from 3 experiments and are normalized to control.

### Transfection

Primers used for human claudin-5 amplification were as follows: forward 5′-TAGTTGAGCTCTGCCAGAGGCTCTGTGATTG-3′; reverse 5′-CGAAGAATTCAGCGCCCTCAGACGTAGTT-3′. Claudin-5 was amplified by PCR from human cDNA using Phusion High-Fidelity PCR Kit (New England Biolabs) according to the manufacturer’s instructions. Both a pEGFP-C2 plasmid (Clontech) and the PCR product were cut by SacI and EcoRI and DNA ligation was performed using T4 DNA Ligase (New England Biolabs). Plasmids were transformed into DH5α *E. coli* and transformed bacteria were selected by kanamycin. Colonies were screened by PCR and the plasmid was verified by sequencing.

For over-expression experiments, cells were transfected using the Electro Square Porator™ according to the manufacturer’s protocol (Protocol 0394). Transfection efficiencies for both eGFP and claudin-5-GFP plasmids were greater than 60%.

**Figure 6:**
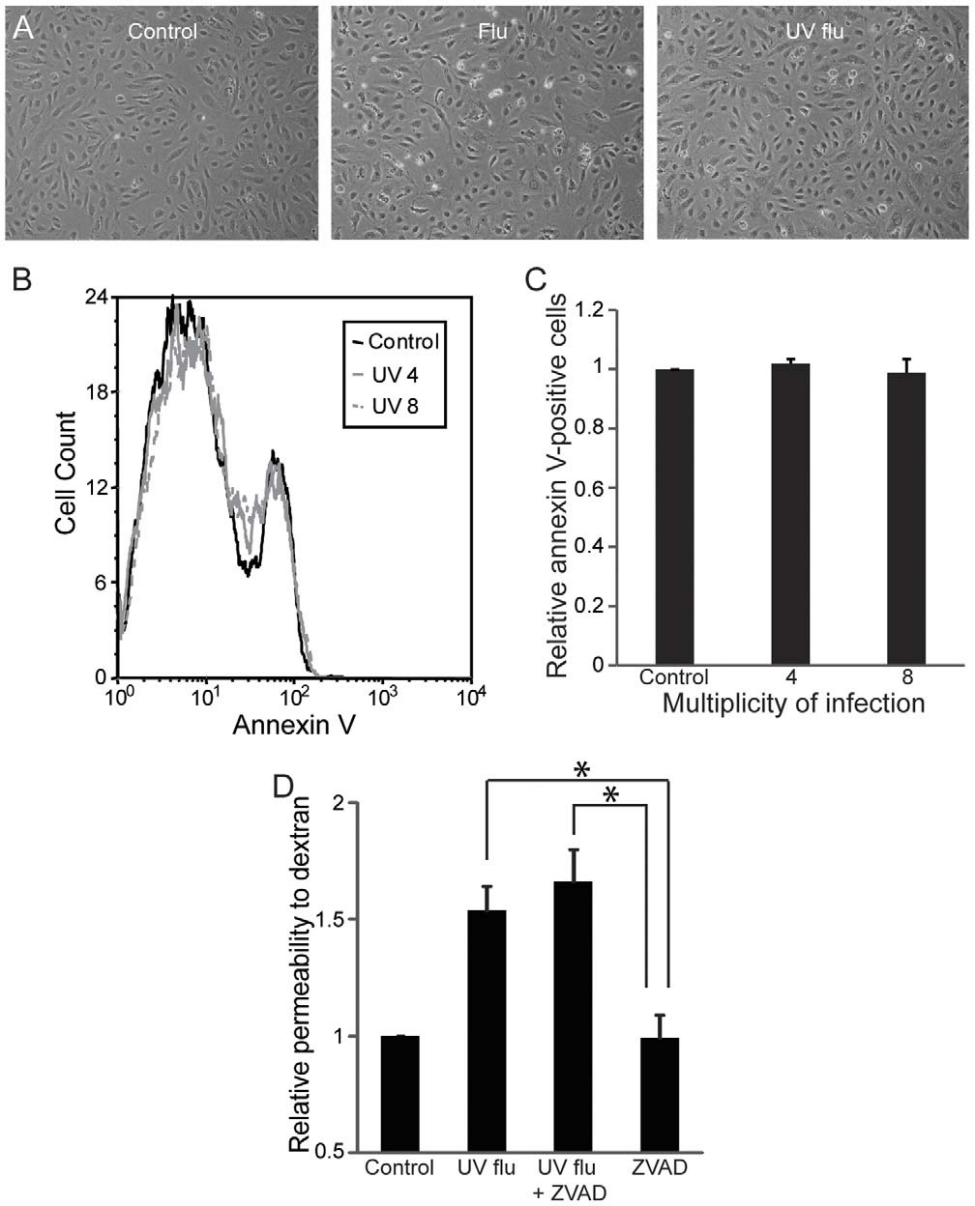
Replication-deficient influenza does not induce apoptosis. (A) Lung endothelium was infected with live or replication-deficient influenza (MOI 8) for 24 hours and then visualized by phase contrast microscopy. Cells exposed to replication-deficient influenza appear much healthier than cells infected with live virus and similar to infected cells. Images were captured as above and are representative of 3 experiments. (B–C) Endothelial apoptosis after exposure to replication-deficient influenza at the indicated MOI was assessed by binding of Annexin V 24 hours later. Histogram (B) is representative of 5 experiments. The percentage of Annexin V-positive cells (C) was similar in all groups. Results (mean, SE) are from 5 experiments. (D) The caspase-inhibitor ZVAD-FMK does not attenuate endothelial permeability induced by replication-deficient influenza. Cells were infected with UV flu (MOI 8) with or without 80 µM ZVAD-FMK for 24 hours. Permeability to dextran was measured. Results (mean, SD) are representative of 3 experiments, *p<0.05.

### Mouse Model of Severe Influenza

C57BL/6 mice were inoculated intranasally with X31 influenza (128 HAU/mouse or 256 HAU/mouse, dose based on pilot data) and lung vascular leak was assessed 4 days after infection. In some experiments, formoterol 0.2 mg/kg or vehicle control was injected intraperitoneally immediately after infection and daily after that. Vascular leak was assessed by quantifying Evans blue dye leak [30], [31]; Evans blue (EB) dye binds tightly to albumin and is a reproducible and accurate means to assess vascular permeability [32]. Ten minutes prior to euthanasia, 100 µL of 1% EB was injected via tail vein. 200 µL of whole blood was collected for EB measurement. The thorax was opened and the mouse perfused with 10 mL PBS to flush the vasculature, after which the lungs were harvested. EB was extracted from tissue by incubation in formamide and absorbances at 620 (A620) and 740 nm were recorded. EB content was calculated by correcting A620 for heme and converted to µg EB by comparing to a standard curve.

**Figure 7:**
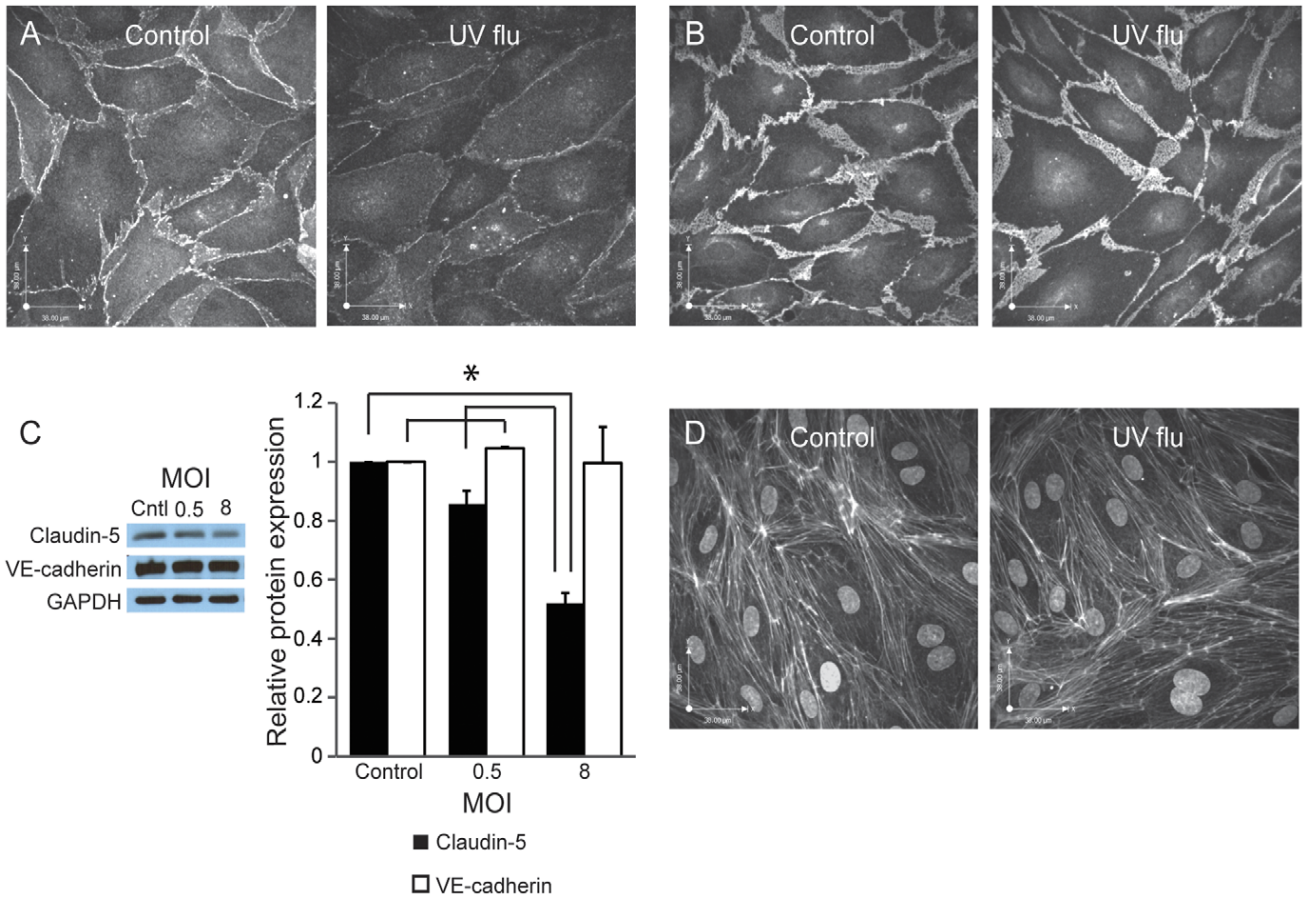
Replication-deficient virus induces loss of claudin-5 while VE-cadherin and F-actin are unaffected. (A,C) Replication-deficient virus induces a loss of claudin-5 as shown by immunofluorescence (A) and western blot (C). In A, cells were infected (MOI 8) for 24 hours. Images are randomly selected and representative of 3 experiments. In C, cells were infected with influenza at the indicated MOI (control  = 0). Image is representative of 4 experiments; histogram is the quantitation and shows mean and standard deviation and is normalized to control, *p<0.05. (B) In contrast, replication-deficient virus (MOI 8 in B) does not affect levels or distribution of VE-cadherin as shown by immunofluorescence (B) and western blot (C). Immunofluorescence images are representative of 3 experiments and western blot is from 4 experiments. The histogram is normalized to control. (D) Replication-deficient virus (MOI 8) does not alter the actin cytoskeleton as shown by immunostaining of F-actin with phalloidin. Images are representative of 3 experiments.

**Figure 8:**
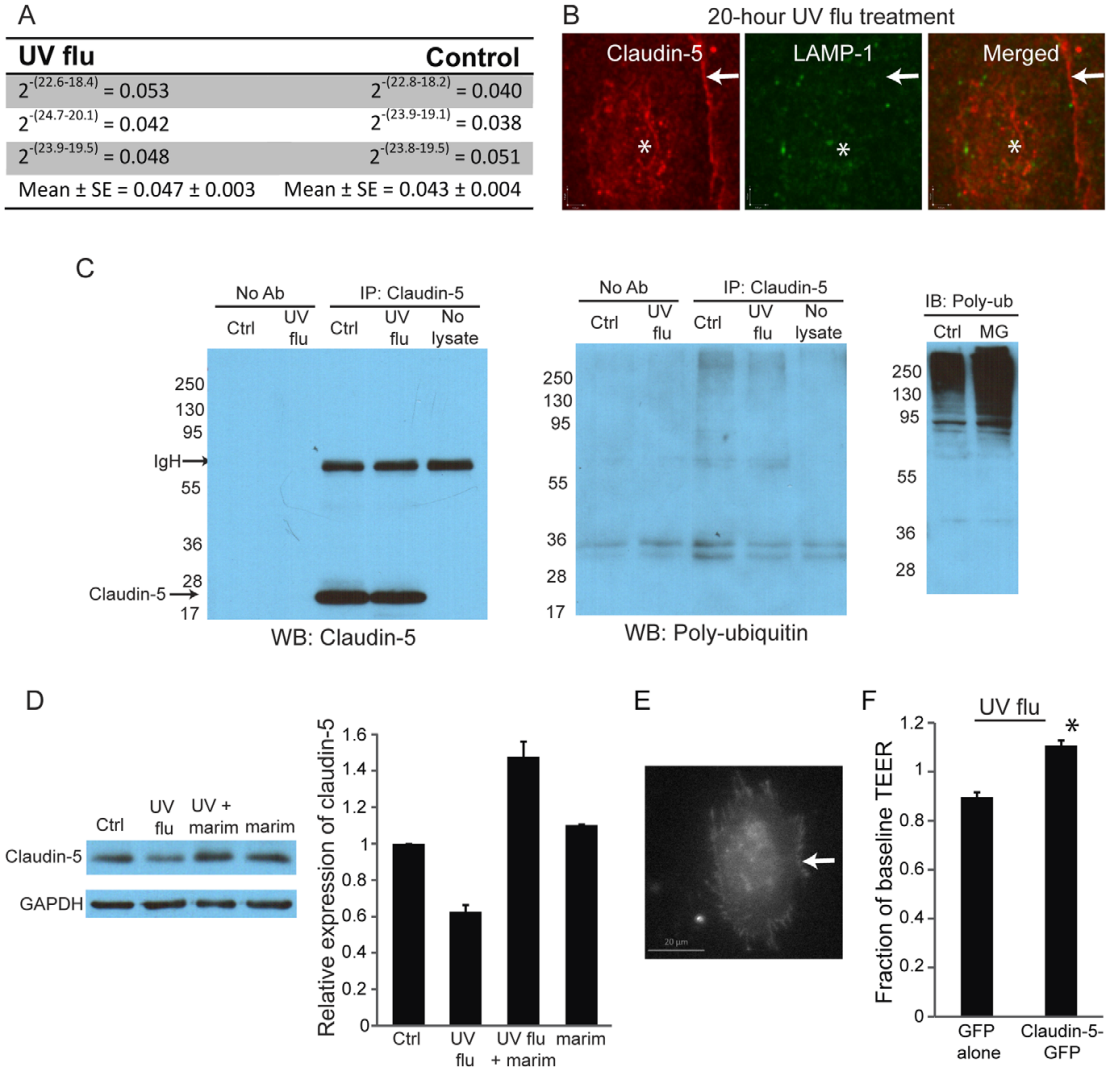
Influenza-induced loss of claudin-5 is due to cleavage by matrix metalloproteases. (A) The reduction in claudin-5 levels is not due to decreased transcription. Levels of claudin-5 mRNA were assessed by quantitative PCR 24 hours after infection with replication-deficient influenza (MOI 4). Results are presented using the comparative C_T_ method using 18S rRNA as a control. Results are from 3 experiments, p>0.05 for control versus infected. (B) Claudin-5 does not co-localize with lysosomes. Cells were infected with UV-irradiated influenza (MOI 8) for 16, 20, and 24 hours and were co-immunostained for LAMP1 and claudin-5. Images were from the 20-hour timepoint, but are reflective of all timepoints. Asterisks indicate cell nucleus, while arrows indicate cell membrane. Images are representative of 3 experiments. (C) Claudin-5 does not interact with poly-ubiquitin. The right-most blot shows the efficacy of the poly-ubiquitin antibody (FK1) since inhibition of the proteasome by MG-132 (MG) increases the expression of poly-ubiquitinated proteins in whole cell lysates. Cells were incubated with UV-influenza (MOI 8) for 24 hours. Whole cell lysates were immunoprecipitated for claudin-5 and blotted for poly-ubiquitin. IgH denotes the heavy chain of the IP antibody. Blots are representative of 3 experiments. (D) Inhibition of MMPs by marimastat blocks influenza-induced loss of claudin-5. Whole cell lysates were probed for claudin-5. Cells were treated with marimastat and infected with UV-flu (MOI 8) for 24 hours. Blot and quantitation are representative of 3 experiments. (E-F) Over-expression of claudin-5 blocks influenza-induced leak. Cells were transfected with either GFP or claudin-5 GFP. Arrow denotes membrane expression of claudin-5-GFP (A). Monolayers were then infected with UV-irradiated influenza (MOI 8) for 24 hours and the TEER was measured pre- and post-infection. Data are mean, SE for 3 experiments, *p<0.05.

### Statistical Analysis

All experiments were performed at least 3 times unless otherwise indicated. Data are expressed as mean and standard error. Student’s t-tests and ANOVAs were used as appropriate. A p value <0.05 was considered significant.

## Results

### Influenza can Infect and Replicate in Human Lung Microvascular Endothelium

We first established that human influenza can replicate in primary human pulmonary microvascular endothelium. We measured the titer of virus over time in the supernatant of endothelial cells in culture and observed a ten-fold increase in viral titer over 24 hours (Fig. 1A). We then measured the replication of viral RNA in endothelium by quantitative PCR and observed a 23-fold increase at 6 hours and a massive increase at 24 hours (Fig. 1B). Finally, we examined expression of viral nucleoprotein (NP) by immunofluorescence of infected endothelium. At 24 hours, approximately 12% of cells were infected (Fig. 1C, 1D). At later time points, expression of NP and viral titer declined (not shown) but the interpretation is confounded by progressive endothelial cell death. Thus, our data convincingly demonstrate that human influenza can rapidly infect and replicate in primary human lung microvascular endothelium.

**Figure 9:**
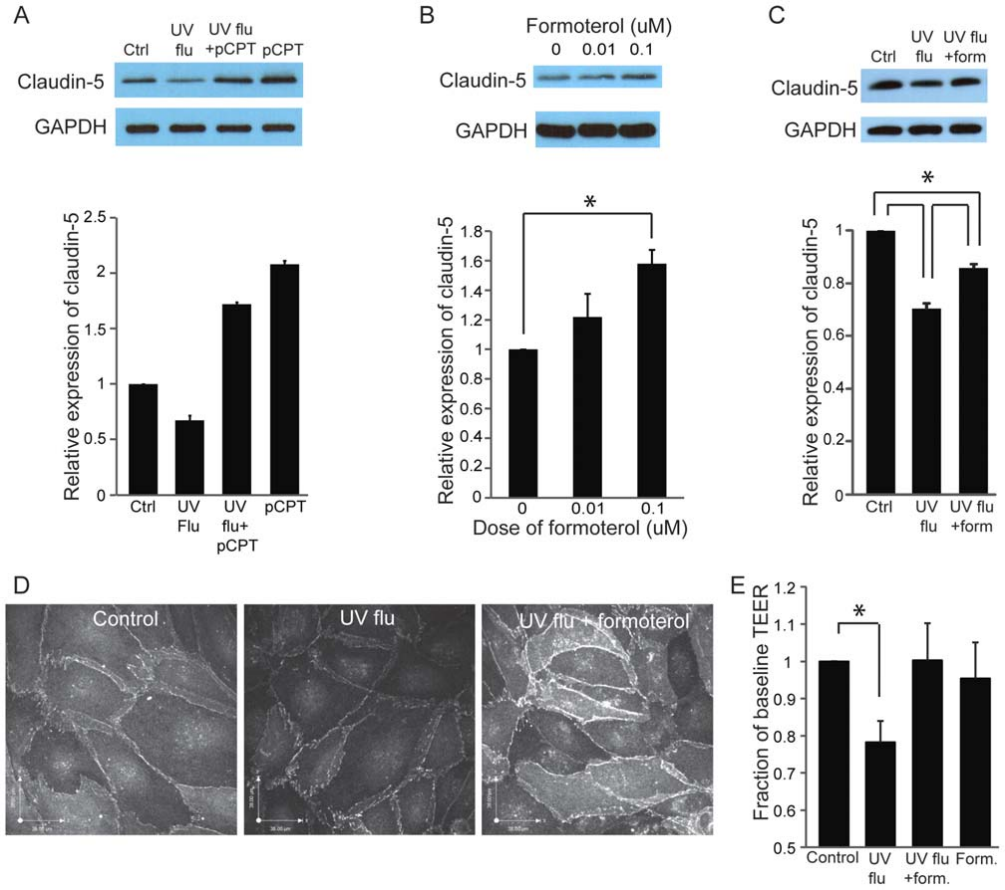
Formoterol induces claudin-5 protein expression and prevents virus-induced endothelial permeability. (A) Whole cell lysates were probed for claudin-5 after incubation with pCPT-cAMP (pCPT, both 24 hours before and during infection with UV-influenza, MOI 8). Blots and quantitation are representative of 5 experiments. (B) Whole cell lysates were probed for claudin-5 after 24 hours of incubation with formoterol. GAPDH is the loading control. Quantitation of claudin-5 protein levels in whole cell lysates, normalized to control (mean and SE); blot is representative of 3 experiments, *p<0.05. (C–D) Formoterol attenuates the loss of claudin-5 induced by replication-deficient influenza. Lung endothelium was infected with replication-deficient influenza (UV-flu, MOI 8) with or without 0.1 µM formoterol. Cells were probed by Western blot for claudin-5 in C and by immunofluorescence in D. In D, note the paucity of claudin-5 at cell-cell junctions of infected cells compared to uninfected controls and the induction of the protein by formoterol. The quantitation in C (mean, SD) is normalized to control, and is representative of 3 experiments; *p<0.05. (E) Formoterol attenuates the endothelial permeability caused by replication-deficient influenza (UV-flu). Cells were infected with UV-flu (MOI 8) with or without 0.1 µM formoterol for 24 hours and permeability was assessed by TEER. Results (mean, SE) are normalized to uninfected cells and are from 5 experiments; *p<0.05.

### Infection of the Microvascular Endothelium causes Increased Permeability Due to Apoptosis

Using a transwell assay in which confluent lung endothelial cells form a tight monolayer over a semi-permeable membrane [28], we observed a dose-dependent increase in permeability of lung endothelium to high-molecular weight dextran after infection with influenza, including with a clinical H3N2 isolate (Fig. 2A, 2B). This was accompanied by a significant drop in transendothelial electrical resistance (TEER) (Fig. 2C), indicating that permeability to both ions and macromolecules was increased by viral infection. The effect of the virus was independent of cell polarity, since infection of endothelial monolayers from the apical or from the basolateral side both induced permeability (Fig. 2D). Infection of the endothelium led to a dose-dependent loss of cell viability that was apparent even under phase contrast microscopy (Fig. 3A). Influenza induced a marked increase in endothelial apoptosis as determined by flow cytometry for Annexin V (Fig. 3B, 3C) with no significant change in necrosis as determined by flow cytometry for propidium iodide (data not shown). The contribution of apoptosis to endothelial leak was determined using the pan-caspase inhibitor ZVAD-FMK [33], since caspases are required for apoptosis [34]. Incubation with ZVAD-FMK greatly prevented virus-induced leak, although the degree of protection was not complete (Fig. 3D).

**Figure 10:**
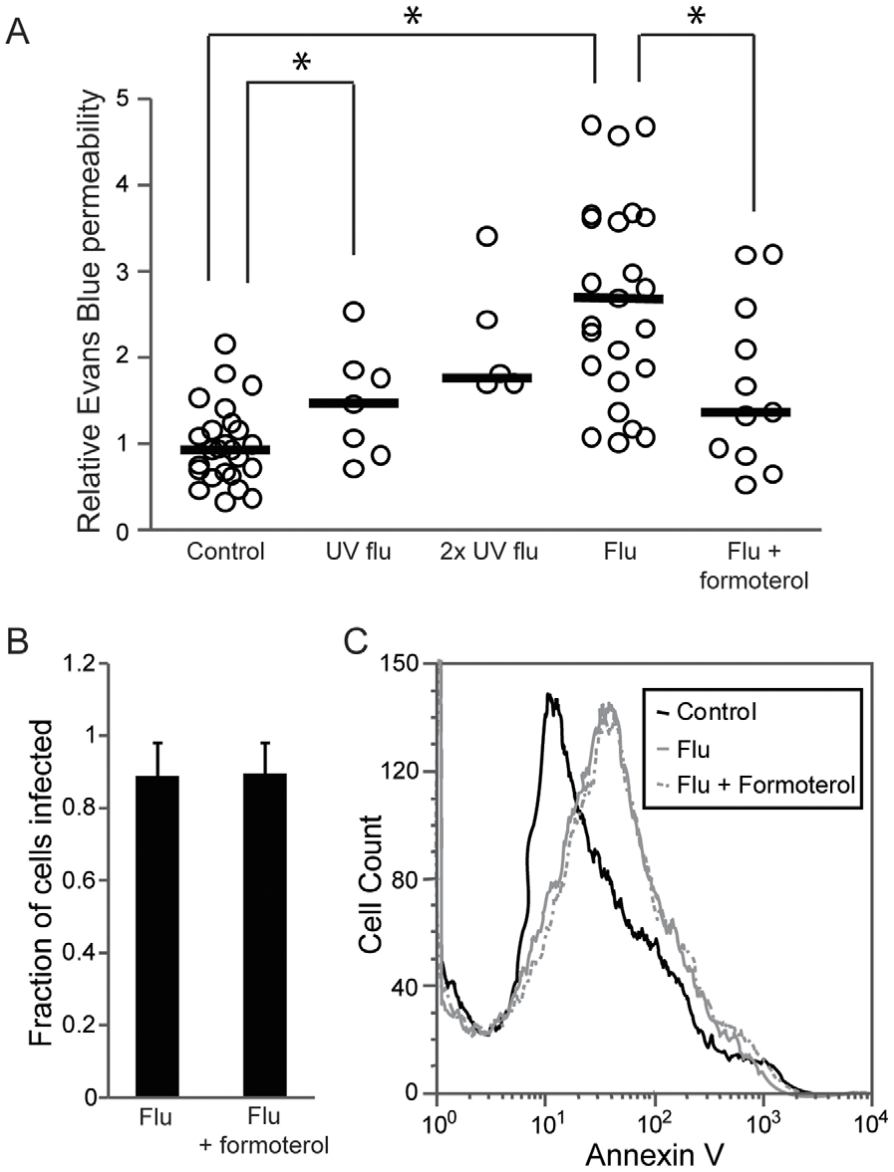
Human influenza causes lung vascular leak in a murine model, which is abrogated by formoterol. (A) C57BL/6 mice were infected nasally with UV influenza (128 HAU/mouse and 256 HAU/mouse), and live human influenza (128 HAU/mouse). Some mice infected with live influenza were given formoterol (0.2 mg/kg) or vehicle by intraperitoneal injection immediately afterwards and daily for 3 days. Lung vascular leak was measured by injection of Evans Blue (EB). Results are means normalized to serum EB levels (to control for unequal administration) and to the mean control EB of the experiment, *p<0.05. (B) Replication of influenza (MOI 8) in mouse lung endothelial cells (with or without 0.1 µM formoterol) was assessed by immunofluorescence for viral nucleoprotein as in Figure 1. Data are mean and SE from 3 experiments. (C) The reduction in lung permeability from formoterol is not due to decreased endothelial apoptosis. Mouse endothelial cells infected with live influenza (MOI 8) with or without formoterol (0.1 µM) were probed with Annexin V 24 hours later. Histogram is representative of 3 experiments.

### Replication-deficient Virus Induces Vascular Leak Independently of Apoptosis

The induction of apoptosis by influenza confounded our ability to interpret any changes in cytoskeletal or junctional processes. To separate out the contribution of viral replication (which might lyse or damage the endothelium) from virus-initiated cellular signaling, we generated replication-deficient virus using ultraviolet light (UV) and confirmed the efficacy of UV-inactivation (Fig. 4A). Cells infected with replication-deficient virus still expressed viral proteins (Fig. 4B) and retained the ability to activate NF-κB (Fig. 4C–D). Remarkably, replication-deficient virus remained capable of inducing endothelial permeability, although to a lesser degree than live virus (Fig. 5A), an effect also observed at varying multiplicities of infection (data not shown). Binding of the virus was not sufficient to induce permeability as treatment of the cells with even high doses of recombinant hemagglutinin (HA) did not cause leak (Fig. 5B). Furthermore, the increase in permeability appeared to be independent of cell death as replication-deficient virus did not induce apoptosis (Fig. 6A–C) or necrosis (data not shown). More importantly, in contrast to live virus, the caspase inhibitor ZVAD-FMK was ineffective at blocking the increase in endothelial permeability that was induced (Fig. 6D). Taken together, these data suggest that influenza mediates lung endothelial permeability by both apoptosis-related and apoptosis-independent mechanisms.

### Replication-deficient Virus Induces Loss of Claudin-5

To elucidate the apoptotic-independent mechanisms for lung endothelial leak, we took advantage of replication-deficient (UV-irradiated) influenza. Replication-deficient influenza caused a significant and dose-dependent reduction in claudin-5 levels in the endothelial monolayer, as assessed by both immunofluorescence and immunoblotting (Fig. 7A, 7C). The loss of the tight junction protein was specific, since levels and the cellular distribution of the adherens junction protein VE-cadherin were unaffected (Fig. 7B, 7C). The increased permeability from replication-deficient virus was also not due to cytoskeletal remodeling, as we could detect no change in either cortical actin or actin stress fibers (Fig. 7D). The decrease in claudin-5 expression was not due to inhibition of protein transcription, since mRNA levels of claudin-5 were unaffected by the virus (Fig. 8A). Over the 24 hours after infection, claudin-5 could not be detected in lysosomes (Fig. 8B), and we detected no colocalization of poly-ubiquitin and claudin-5 by immunofluorescence (data not shown). We also found no interaction of poly-ubiquitin and claudin-5 by immunoprecipitation (Fig. 8C), suggesting that claudin-5 was not being degraded by the proteasome. Instead we found that inhibition of matrix metalloproteases (MMPs) by the broad-spectrum MMP inhibitor marimastat [35] was sufficient to block the loss of claudin-5 (Fig. 8D), suggesting that the increased loss of claudin-5 was due to extracellular cleavage by MMPs. To prove that the loss of claudin-5 is required for replication-deficient virus-induced permeability, we over-expressed claudin-5-GFP in the lung endothelium and verified that it was appropriately targeted to the plasmalemma (Fig. 8E). While control (GFP-transfected) endothelium continued to exhibit increased leak after exposure to the virus, claudin-5-GFP-expressing monolayers did not (Fig. 8F). Thus, over-expression of claudin-5 is sufficient to block replication-deficient influenza-induced leak.

### The Beta_2_ Agonist Formoterol Attenuates Virus-induced Leak and causes Induction of Claudin-5

Beta_2_ agonists are well-described to have vascular barrier-enhancing effects that are attributed to increases in cellular cAMP; these include the induction of claudin-5 [36]. We treated lung endothelium with an analog of cAMP, pCPT-cAMP, and confirmed that claudin-5 expression was induced (Fig. 9A). We also noted that formoterol could induce claudin-5 protein levels in a dose-dependent fashion (Fig. 9B). Furthermore, formoterol attenuated the loss of claudin-5 provoked by replication-deficient influenza (Fig. 9C), leading to enhanced protein expression at the cell membrane and in intracellular organelles (Fig. 9D). Finally, formoterol significantly attenuated endothelial permeability induced by replication-deficient virus (Fig. 9E).

### Influenza Induces Pulmonary Edema in a Mouse Model that is Attenuated by Formoterol

To validate our in vitro observations, we created an experimental model of influenza in mice. Both live and UV-irradiated human influenza induced significant pulmonary edema within days after infection; in keeping with our in vitro data, the effect of live virus was greater. Parenteral treatment with formoterol essentially abrogated virus-induced lung vascular leak (Fig. 10A). As controls, we first determined that this was not due to impairment by the drug of viral replication by probing infected mouse endothelial cells for viral nucleoprotein. Formoterol-treated endothelium had no significant decrease in the expression of viral nucleoprotein, indicating that viral entry, viral replication and viral protein expression are all unaffected by the drug (Fig. 10B). We also ruled out inhibition of virus-mediated endothelial apoptosis as the mechanism of formoterol’s protective effect (Fig. 10C). We obtained the same results using human lung endothelium (data not shown). These findings are thus consistent with the notion that enhancement of the vascular barrier by formoterol, including its induction of claudin 5, attenuates influenza-mediated lung injury.

## Discussion

While influenza usually causes a self-limiting illness, the most severe infections require admission to an intensive care unit for respiratory failure. There, patients require mechanical ventilation for days to weeks and exhibit a mortality rate of close to 20% [2], despite antiviral drugs and supportive care. Respiratory deterioration in severe influenza is due to acute lung injury [2], a syndrome in which the pulmonary microvasculature leaks leading to alveolar flooding and profound hypoxemia. The pathogenesis of this complication remains unclear [37], but the *sine qua non* of the syndrome is increased lung microvascular permeability. The endothelial barrier is a critical component of the alveolar-capillary membrane that normally prevents pulmonary edema, and abundant data indicate that alterations or damage to the endothelial monolayer are sufficient to induce microvascular leak [20].

Remarkably, the possible contribution of the lung endothelium to the pathogenesis of severe influenza has been largely neglected. Previous reports using umbilical vein endothelium have described cytokine production after infection with human influenza [38] or extravasation of albumin from the organs of animals infected with flu [4]. However, to date, no-one has examined the effect of infection *per se* on lung microvascular leak. Furthermore, the use of umbilical vein endothelium in previous studies is of questionable relevance given the established heterogeneity of endothelial tissue beds [39] and the primary importance of microvascular (as opposed to large-vessel) lung leak during ALI.

We found that human influenza dramatically increased apoptosis of the lung endothelial monolayer, leading to decreased endothelial viability and a marked increase in endothelial permeability. The increased permeability was independent of endothelial cell polarity, indicating that such leak could easily occur in vivo due to the proximity of the respiratory epithelium. The fact that barrier integrity was at least partially restored when caspase activity was inhibited implies that apoptosis is the cause rather than the consequence of the increase in permeability. To separate out the contribution of viral replication (which might lyse or damage the endothelium) from virus-initiated cellular signaling, we generated replication-deficient virus using ultraviolet light. In addition to being a useful tool, replication-deficient virus reflects clinical reality in which treatment with antiviral drugs is only partially effective at reducing mortality [40]. This incomplete protection may suggest that inhibition of the viral life cycle does not completely prevent damage to the human host. Accordingly, we observed that replication-deficient virus can induce significant lung endothelial permeability, without causing endothelial apoptosis or necrosis. Thus, virus-induced endothelial apoptosis is sufficient but not necessary for lung endothelial permeability. Instead, we observed a dose-dependent loss of claudin-5, an endothelial-specific protein enriched in the lung that is the major constituent of intercellular tight junctions.

To date, most literature has instead focused on alterations in adherens junctions, usually culminating in the internalization and/or degradation of VE-cadherin [41]. We observed no effect of human influenza on the distribution or the amount of VE-cadherin by immunoblotting or immunofluorescence. This indicates that the loss of claudin-5 is not due to non-specific degradation of junctional proteins and that dismantling of tight junctions can occur in the presence of apparently normal adherens junctions. Furthermore, the lack of apoptosis in cells infected with replication-deficient virus indicates that the loss of claudin-5 is not simply a byproduct of cell death.

In principle, the decrease in claudin-5 protein after viral infection could represent an impairment of protein synthesis and/or an increase in its degradation. We observed no effect of the virus on claudin-5 mRNA levels, ruling out diminished transcription as the cause of the reduction in protein. Instead, our data suggest that influenza induces endothelial permeability by stimulating the degradation of claudin-5 from the cell surface by matrix metalloproteases. Although lysosomal degradation of claudins has been described [42], we observed no colocalization of claudin-5 with the late endosome/lysosome marker LAMP1 [43] over 24 hours. Similarly, while a role for the ubiquitin-proteasome system in claudin-5 metabolism was recently reported [42], we detected no increase in ubiquitination of the protein after viral infection. We were unable to assess the effect of proteasome inhibition on claudin-5 loss, as proteasome inhibitors proved toxic to virally-infected cells. However, the fact that inhibition of matrix metalloproteases was sufficient to restore claudin-5 levels strongly supports a role for MMP in the loss of claudin-5. Because marimistat is a broad-spectrum MMP inhibitor, further work is necessary to identify the specific metalloprotease(s) responsible for claudin-5 degradation.

While we have focused on the role of cell-to-cell junctions, the actin cytoskeleton is known to be an important player in the regulation of the endothelial barrier [28]. The formation of actin stress fibers induces endothelial cell contraction and increased permeability; in contrast, cortical actin formation at cell borders enhances barrier integrity. Both processes are tightly regulated by members of the Rho GTPase family [20]. Although we did not see any change in the actin cytoskeleton by immunofluorescence, further studies should determine whether influenza-mediated permeability involves RhoGTPases or actin remodeling.

An important question clinically is how to improve the outcome of ALI from influenza. In vivo, endothelial infection, leukocyte adhesion, and other factors such as systemic cytokines and the release of leukocyte granules [44] may synergize to induce lung injury. Thus, there is considerable interest in developing therapeutic agents that will protect the ultimate downstream target - the endothelial barrier [45] - even in the face of a multifactorial insult. Unlike antiviral drugs, this therapeutic approach targets the endothelium rather than the pathogen and should have the advantage of being less susceptible to viral mutation. Beta_2_-adrenergic agonists such as formoterol stimulate intracellular adenyl cyclase leading to increased intracellular cAMP, which is known to have a multitude of vascular barrier-enhancing effects [46] including the induction of claudin-5 [36], [47]. There has been considerable interest in these clinically-approved drugs in the treatment of lung injury; however, human trials with beta_2_-agonism in pulmonary edema have shown opposing results [48], and one recent trial was stopped early due to harm [49]. However, in that trial of patients with acute respiratory distress syndrome, only one dose of the drug was tested and the mortality rate in the control group was much lower than expected. In addition to possible toxic effects on non-vascular tissues, another factor that may have contributed to the conflicting results is that the effect of cAMP on endothelial barrier integrity varies according to its subcellular location. While cAMP near the plasma membrane enhances barrier integrity, cytosolic cAMP is barrier-disruptive [46]. Thus, it is possible that different doses of beta_2_-agonists might be protective in lung injury. In our in vivo model, formoterol attenuated the induction of lung vascular leak by human influenza, although interpretation of the mechanism of benefit is limited by the lack of specificity of the drug. Further work is ongoing in our lab to determine whether formoterol and other more specific vascular-enhancing agents can improve the clinical outcome from severe influenza.

We have identified two distinct mechanisms by which influenza may induce pulmonary edema. The fact that mortality is not eliminated in patients who receive antiviral drugs [40](which suppress viral replication) is consistent with our data demonstrating that replication-deficient virus can still degrade the endothelial barrier. The relationship, if any, between endothelial apoptosis and degradation of claudin-5 is unclear at present. However, the fact that replication-deficient influenza did not induce endothelial apoptosis indicates that the loss of claudin-5 is not a consequence of cell death. It is possible that these two mechanisms collaborate in vivo to further aggravate endothelial leak, with infected cells undergoing apoptosis while adjacent cells, which might be exposed to less replicating virus, would still undergo degradation of claudin-5. Ultimately, however, examination of each mechanism in vivo will be required to determine their relative contribution to influenza-mediated acute lung injury.

In summary, we describe two distinct potential mechanisms for the pathogenesis of ALI after infections with human influenza. Infection of the pulmonary endothelium can lead to endothelial apoptosis and microvascular leak; furthermore, even replication-deficient virus can promote the loss of claudin-5, a critical component of endothelial tight junctions (Fig. 7). These processes may culminate in marked deficiency of the alveolar-capillary barrier, leading to alveolar flooding and hypoxemia. These findings thus raise the possibility that enhancement of lung endothelial barrier function may constitute a novel therapeutic approach for severe human influenza.
